# Implementing active surveillance for tuberculosis: A quality improvement project

**DOI:** 10.4102/safp.v67i1.6106

**Published:** 2025-05-29

**Authors:** Febisola I. Ajudua, Robert Mash

**Affiliations:** 1 Division of Family Medicine and Primary Care, Faculty of Medicine & Health Sciences, Stellenbosch University, Cape Town, South Africa; 2 Department of Family Medicine and Rural Health, Faculty of Health Sciences, Walter Sisulu University, Mthatha, South Africa; 3 Department of Family Medicine, Faculty of Health Sciences, Nelson Mandela University, Gqeberha, South Africa

**Keywords:** active surveillance, TB, tuberculosis, community health worker, community-oriented primary care, COPC, community-based services

## Abstract

**Background:**

South Africa is a high tuberculosis (TB)-burden country with the worst multidrug-resistant TB (MDRTB) epidemic in Sub-Saharan Africa. The recommendations of the World Health Organization (WHO) in high TB-burden settings are to institute processes for identifying patients with active TB and to improve social support. The community-oriented primary care (COPC) model relies on the community health workers’ (CHW) every encounter in the community as an opportunity to screen for TB symptoms. This study aimed to evaluate the implementation of active surveillance for TB in a CHW team.

**Methods:**

This was a quality improvement project (QIP) focused on the implementation of TB screening in the community-based services at a primary care facility in the Nelson Mandela Bay Health District (NMBHD).

**Results:**

The baseline audit revealed one team was available in the facility even though it serviced two and a half municipal wards. The team comprised an outreach team leader and three CHWs. There were no records of community-based TB screenings done. The midway audit showed a remarkable rise in clients screened in the community. There was a failed attempt to introduce the use of mHealth technology to the team. The audit at the end of the QIP showed a continuing lack of adequate records of activities in the community.

**Conclusion:**

The CHWs in this study, although capable and motivated, lacked opportunity to perform adequate community-based TB screening because of the lack of supportive supervision, inadequate recordkeeping, and a district managerial team that focused on the practice population rather than the population at risk.

**Contribution:**

We recommend a continuing QIP and a re-education of health care providers about community-based health services.

## Introduction

Tuberculosis (TB) is a leading cause of morbidity and mortality globally.^1^ It ranks eighth in the top 10 causes of death in Africa.^2^ South Africa is a high TB-burden country and has the worst multidrug-resistant TB (MDRTB) epidemic in Sub-Saharan Africa.^3^ The causes for the South African epidemic are multifactorial.^4,5,6^ The predominant factors being the prevalence of the human immunodeficiency virus (HIV) and the contribution of social drivers in poor areas. These factors, and inadequate access to health care for the socioeconomically disadvantaged population, make it difficult to control the epidemic.^7^ It is important to highlight here that difficult access is not always because of geographical or economic factors, but more complex factors such as health-seeking behaviour and belief systems.^8,9^

The recommendations of the World Health Organization (WHO), in settings with a high burden of TB, include a focus on diagnosis of all active TB, a strengthened capacity for linkage to care for patients suspected of active TB, and initiatives that provide social support to these patients.^10^ The need for social support to mitigate the catastrophic effects of the disease is shown by the positive effect of incentives on adherence to therapy.^11,12^ The National Tuberculosis Programme (NTP), prioritises early identification of active TB in patients through active surveillance.^13^ In line with WHO recommendations, the NTP targets populations with a high risk for TB.^14^ This includes persons living in poor areas and urban slums, where the prevailing social drivers of the TB epidemic such as poverty, unemployment, malnutrition, overcrowding, and poor living conditions, combined with multimorbidity, predispose to TB disease.^15^

The South African district health system has, at its foundation, primary health care, including community-based health services.^16^ One of the main strategies is the introduction of community health workers (CHWs), as part of ward based primary health care outreach teams (WBPHCOTs). They are trained to provide screening services in communities for the early identification of both communicable and non-communicable diseases. Active surveillance for TB is one of the many services provided by CHWs. Active surveillance is defined as all activities conducted by health care workers in the community to identify previously undetected active cases of TB.

The South African population is estimated to have a TB incidence of 554/100 000 population and reports confirm that there are communities where the incidence is even higher.^17,18^ The health system has a quadruple burden of disease in a resource-limited context.^19^ Any activities responding to the epidemic, as recommended by the WHO, should consider the availability of resources in the health system to respond adequately to new cases identified by active surveillance. There is evidence supporting the use of active surveillance for TB in settings where there is a high burden of TB.^10,20^ However, active surveillance for TB as a stand-alone strategy is associated with costs that may not be sustainable in resource limited settings.^21,22^

The END TB goals aimed to reduce morbidity because of TB by 90% in 2035.^23^ Several approaches have been recommended to achieve these goals, including the use of preventive measures to reduce the incidence of TB in communities.^24^ Some of these approaches include ensuring universal health coverage with the use of TB screening and improved access to diagnostic tools.^25^ Universal health coverage is a goal of the National Department of Health (NDoH) to address the quadruple burden of disease, which includes TB. It also recognises the need to collaborate with social services.^11^ Community-based interventions are often a collaboration between stakeholders, including the NDoH and other government departments responsible for social development, and non-profit organisations (NPOs).

Implementing active surveillance for TB requires capable and motivated health care workers. Community health workers undergo training before entry into the community for service delivery.^15^ Their training requires an understanding of the TB epidemic in communities, with the need for improved access to health services for clients with TB symptoms. Implementing active surveillance also requires the motivation of CHWs to take the available opportunities and link clients to care.^24^ It is important that the process of screening and referral is recorded to allow monitoring and evaluation of performance.

Capable and motivated CHWs also require a service delivery environment that enables them to take advantage of the opportunities for active surveillance. Community-oriented primary care (COPC) is the current model of care that shifts service delivery from a focus on facility-based primary care to a focus on comprehensive primary health care (PHC).^25^ In the SA context, this usually involves community-based WBPHCOTs who must work with the facility-based staff in one multidisciplinary PHC team. This model of care relies on the CHW to see every encounter in the community as an opportunity to screen people for TB symptoms, while also providing other services. Each CHW has a designated number of households for which they are responsible.^17^ Thus, the whole population can be screened for symptoms of TB and people with undiagnosed TB disease, who are spreading the disease, can be identified. Identification of the patient, presumed to have TB, contributes to reducing the incidence of TB in the community.^26,27^ In addition, CHWs can perform contact tracing, based on patients who have already been diagnosed at the primary care facility.^28,29^ Community health workers must be able to identify those at-risk of active TB from the symptom screen and refer them to primary care services where they can be diagnosed and treated.^30^ Community health workers must document their activities, so that the success of active TB surveillance can be monitored, and hotspots of TB disease identified in the community.^31^ Community health worker teams are supervised and supported in the community by professional nurses, known as outreach team leaders (OTL).^17^ Implementation is also heavily reliant on the acceptance of the CHWs and their work by community members.^32,33^

Despite the many initiatives to control the TB epidemic in South Africa, the Nelson Mandela Metropole has a high incidence of TB and is one of the worse affected health districts in the Eastern Cape.^34^ Previous studies identified key factors that influenced the success of active surveillance for TB.^34^ These factors were often not specific to TB, but related to the performance of WBPHCOTs and implementation of COPC. Training and supervision of the CHWs, was regarded as key, as well as the acceptability of their services by the community. Acceptability was influenced by the ability of CHWs to make a difference, particularly for social needs and services. Security and transport were sometimes a challenge in provision of services. Overall, CHWs required certain soft skills to form relationships with other stakeholders in the community and to engage with and motivate their clients. This study aimed to evaluate the process of implementing active surveillance for TB in an existing WBPHCOT in the Nelson Mandela Bay Health District (NMBHD) and to learn how the quality of these services could be improved.

## Research methods and design

### Study design

**FIGURE 1:**
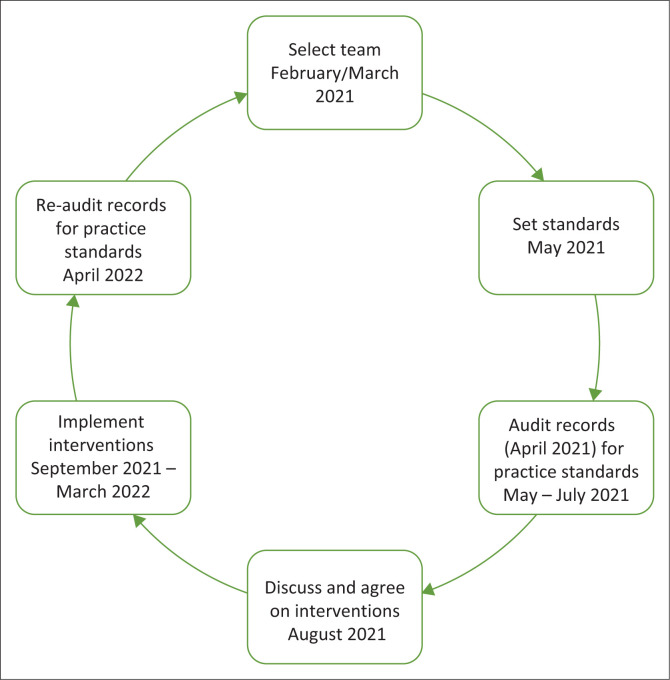
Quality improvement cycle including timelines for the essential steps in the project.

This was a quality improvement (QI) study during 2021–2022 that focused on active surveillance for TB. The study followed the usual steps of the QI cycle (Figure 1): establishment of a QI team, setting of target standards, baseline measurement of performance, reflection on performance, planning and implementing change, re-measuring performance after one year.

### Setting

**FIGURE 2:**
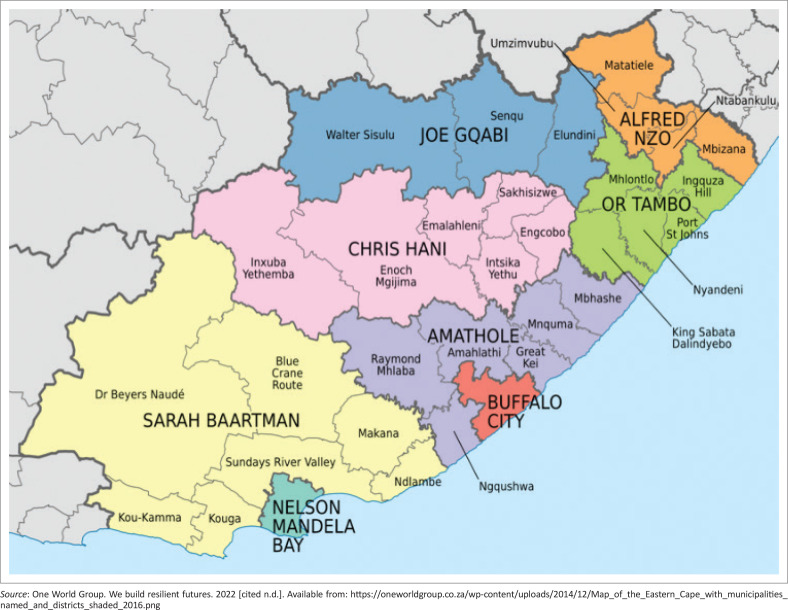
Position of Nelson Mandela Bay District in Eastern Cape province.^53^

**FIGURE 3:**
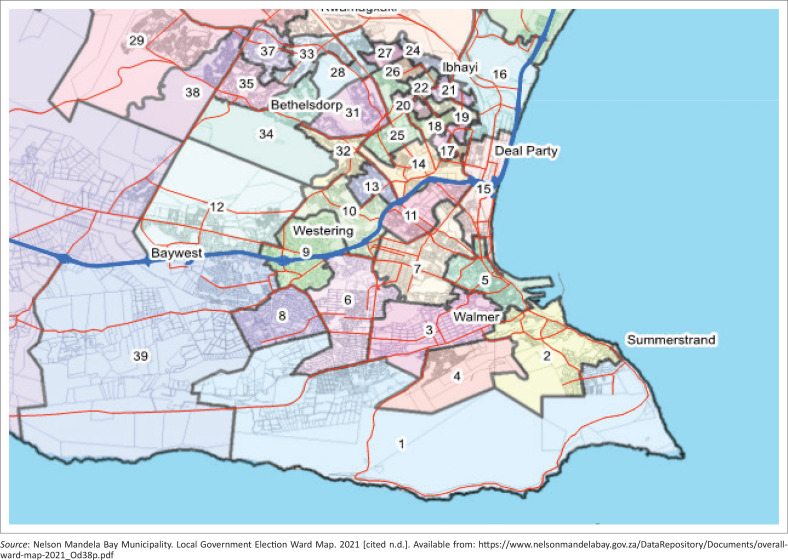
Location of wards 25 and 26 in Nelson Mandela Bay District in Eastern Cape province.^54^

This study was based at a primary care facility in sub-district A of the NMBHD (Figure 2 and Figure 3). The facility served three municipal wards and the CHW team provided services across these wards. The WBPHCOT consisted of three active CHWs and an OTL. The WBPHCOT assisted the TB room nurses with contact tracing for TB patients, and retaining or returning patients to TB therapy. The WBPHCOT provided active case finding for TB in the community as part of their scope of practice by screening clients in their homes during home visits. They also screened clients in taverns or community spaces where they had the opportunity to provide community education. Clients identified to have symptoms of TB were referred to the facility for further investigation.

This community was mainly Xhosa speaking with a high rate of unemployment and a heavy reliance on the public health sector. The housing in the community mainly consists of basic housing with backyard shack dwellers. There was a high rate of crime and social problems such as substance abuse. The PHC facility was situated close to a regional hospital in the sub-district and the team was sometimes required to assist them with tracing TB patients. The facility manager indicated that records of referrals received from the WBPHCOT were a data element in the national Ideal Clinic reporting tool.^35^

### Selecting a team

After a series of meetings in February 2021 to introduce the quality improvement project (QIP) with the WBPHCOT, the TB room nurse at the facility, the facility manager and the service coordinators, an initial meeting was set for May 2021 with the members who had agreed to be part of the QIP (Table 1). At the time of the study, there was no additional NPO providing TB screening services in this community. The NPO that was present focused on tracing patients with MDRTB and linking them back into care. There was one WBPHCOT at the facility and this team’s members joined the QIP team. The facility manager and the TB nurse did not want to participate in the QIP because it concerned community-based services and was seen as extra work that would negatively impact their contribution to the facility. The researcher was a family physician from a local district hospital who facilitated the team meetings and process.

**TABLE 1:** Quality improvement project team including designations, role in the ward based primary health care outreach team and in the quality improvement project team.

Designations	Roles in WBPHCOT	Roles in QIP
Outreach team leader	Provides leadership, support, and supervision to the team	Provide direction to the CHWs, discuss, plan and reflect with team, encourage CHWs to perform tasks, set targets with team, and monitor achievements
Community health workers	Provide community-based health services	Perform tasks set for the WBPHCOT, set targets with team, discuss, and plan and reflect on accomplished tasks
Researcher and/or doctor	Provide expert advice on recommended interventions in community health issues or individual clients	Facilitate the team, set meeting dates, establish set targets with team, discuss plans, monitor achievements, and interpret findings

WBPHCOT, ward based primary health care outreach team; QIP, quality improvement project; CHWs, community health workers.

### Setting target standards

The researcher worked with the QIP team to identify the activities needed for active TB surveillance in the community and set standards based on the structure, processes, and expected outcomes (Table 2). This was done in May 2021. Structure refers to the infrastructure and resources needed; processes refer to the activities of health care workers and outcomes to the expected effects of the activities.

**TABLE 2:** Target standards, data required, and data sources.

Target standards	Data required	Data sources
**Structure**		
The team is headed by an OTL	Number of OTLs	Facility management and/or observation
The team consists of four CHWs	Number of CHWs per team	OTLs
CHWs are trained for TB surveillance	Number of CHWs trained in TB surveillance	OTLs
Stationery for TB surveillance is always available in the last 4 weeks	Availability of household assessment forms	OTLs and/or observation
Stationery for referral and feedback is always available in the last 4 weeks	Availability of referral forms	OTLs and/or facility management and/or observation
**Process**		
CHWs conduct 75% of the expected home visits in the preceding month (five visits per day is the norm)	Number of households registrations and/or home visits in the preceding month	Household registration forms and/or OTLs and/or facility management
CHWs conduct TB symptom screening in 80% of the households visited	Number of households screened for TB and/or number of households visited in the preceding month	Household registration forms and/or screening tools
CHWs refer 80% of the clients who screen positive to the facility using a referral letter	Number of these clients referred to facility for TB and/or number of clients screened positive for TB	Referral slips collected at facility
		Household registration forms and/or screening tools
A total of 80% of the clients referred have feedback on the results of investigation in collection box at the facility	Number of these clients who have feedback provided on investigations and/or number of clients referred to facility for TB	Referral booklet records and/or referral slips collected
A total of 80% of the clients that are referred and attend the facility are investigated for TB	Number of these clients that are investigated and/or number of clients referred by CHWs who attend the facility	TB suspect register
**Outcomes**		
A total of 60% of the population seen is screened for TB	Number of clients screened for TB	Record of screening tools reflecting TB screening done
	Number of clients in designated population	
A total of 60% of the clients who screen positive for TB in the community are investigated at the facility	Number of these clients with evidence of investigation at the facility (NHLS)	TB suspect register
	Number of clients screened positive for TB	
A total of 90% of the clients referred by CHWs that test positive for TB are started on treatment	Number of clients referred by CHWs started on treatment	TB suspect register record
	Number of clients referred by CHWs that test positive for TB	

OTL, outreach team leaders; CHW, community health workers; TB, tuberculosis; NHLS, National Health Laboratory Service.

### Baseline data collection

A baseline audit of the team’s practice standards in April 2021 was carried out from May through July 2021. Data were collected, as described in Table 2, and the findings presented to the QIP team.

### Feedback, planning, and implementation of interventions

The team discussed the findings and planned several interventions to improve active TB surveillance. The team met monthly to monitor progress and implement the interventions over a period of 7 months. Tasks were allocated to team members and feedback given at each meeting. Interventions could be adapted to improve feasibility and other stakeholders identified. An informal audit (not presented in the results) was performed after 6 months to give some feedback on progress.

### Follow-up data collection and analysis

A follow-up audit of the target standards was conducted in April 2022, and the results were compared to the baseline audit of April 2021.

### Reflection on learning

The researcher set up sessions for reflection with the OTL, the CHWs and the subdistrict coordinator of the WBPHCOTs to reflect on the results of the QIP and the underlying factors that impacted these results. Even though the coordinator did not participate in the team, the team considered it essential to understand some of the challenges described during the QIP from the coordinator’s perspective.

These discussions were recorded and on reviewing the recordings, the researcher summarised the key issues identified. The researcher conducted a thematic analysis of the recorded interviews with the coordinator and the OTL. The researcher summarised the data. During the final group reflection of the team, the researcher asked the team about the themes identified in these transcripts to get their perspectives of the main themes identified during the initial interviews with the subdistrict coordinator and the OTL. The QIP team reached a final consensus on the key issues that had influenced the QIP during the group reflection at the end of the cycle.

### Ethical considerations

Ethical approval for this study was received from the Health Research Ethics Committee (HREC) 2 of the Faculty of Medicine & Health Sciences, Stellenbosch University on 16 January 2018 (reference number S17/10/189_1243). Permission for the study was granted by the Eastern Cape Health Department via the National Health Research Database and the office of the manager for district health services in the NMBHD.

## Results

### Baseline audit

The results of the baseline audit are presented in Table 3. Four of the five structural standards were met. None of the process or outcome standards were achieved. Only one WBPHCOT was available even though the facility provided services to two wards and part of a third ward. This team was composed of an OTL and three CHWs. The CHWs were all trained in TB screening prior to starting their duties but had no refresher training in the year preceding the audit. There was no recording of TB screening in any of the records used by the team during home visits. In fact, the tool available for recording home visits had no place to confirm provision of TB screening services. Verbal reporting from the CHWs stated that they were conducting TB screening. There was no formal recording of the TB screening or the referral of clients to the primary care facility.

**TABLE 3:** Baseline and follow-up audit results.

Target standards	Baseline audit	Follow-up audit
**Structure**		
The team is headed by an OTL	Yes	Yes
The team consists of four CHWs	No	No
Community health workers are trained for TB surveillance	Yes	Yes
Stationery for TB surveillance is always available in the last four weeks	Yes	Yes
Stationery for referral and feedback is always available in the last four weeks	Yes	Yes
**Process**		
Community health workers conduct 75% of the expected home visits in the preceding month (five visits per day is norm)	13%	26%
Community health workers conduct TB symptom screening in 80% of the households visited	0%	0%
Community health workers refer 80% of the clients who screen positive to the facility using a referral letter	0%	0%
A total of 80% of the clients referred have feedback on the results of investigation in collection box at the facility	0%	0%
A total of 80% of the clients who are referred and attend the facility are investigated for TB	0%	0%
**Outcomes**		
A total of 60% of the designated population is screened for TB	0%	0%
A total of 60% of the clients who screen positive for TB in the community are investigated at the facility	0%	0%
A total 90% of the clients referred by CHWs who test positive for TB are started on treatment	0%	Not applicable†

OTL, outreach team leaders; CHW, community health workers; TB, tuberculosis.

† , all referred clients were TB negative.

### Interventions planned to improve tuberculosis screening

The QIP team reflected on the baseline audit and planned changes to improve the quality of active TB surveillance:

- The QIP team leader met with the subdistrict coordinator and the facility manager to ask for more CHWs in the facility.
- Community health workers were expected to record TB screening activities to make monitoring and evaluation feasible. The team agreed that all stationery, whether photocopied or printed, was acceptable. Implementation would be monitored for six months to be followed by informally auditing the household registration forms and CHW records.
- Community health workers were expected to start using the available referral forms to link people with suspected TB to care at the PHC facility. It was agreed that the OTL would provide the referral forms to the team.
- The QIP team leader met with the facility manager and subdistrict coordinator to support a functional multi-disciplinary team (MDT) meeting. The intent was to implement an MDT at the facility where facility-based health workers would engage with community-based workers and coordinate care.
- A feedback system of communication between the clinicians and the WBPHCOT would be implemented in the facility for all referrals. The plan was that the nurses in the TB room would provide written feedback on the referrals, which would be collected by the OTL.

### Follow-up audit

The results of the final audit, in April 2022, are presented in Table 3. Again, four out of the five structural target standards were met. No additional CHWs were employed in the WBPHCOT. None of the process standards were achieved, although there was an increase in the number of home visits. In addition, two clients were referred to the PHC facility for TB investigation, but it was not clear how these were identified, as the household forms had no record of TB screening. Both of these clients were investigated, found to be TB negative, and feedback was given to the CHWs. The overall outcomes, therefore, were unchanged.

### Final reflections on the quality improvement project

#### Team composition

There was a shortage of CHWs on the team, which was further compounded by the insufficient supervision and support they received from the OTL, who was only available some of the time.

#### Active tuberculosis surveillance

The CHWs were quite motivated to screen for TB and initially started to record household screening in the forms provided. During this period, however, the department of health introduced m-health technology to improve data collection and household registration by CHWs. Community health workers perceived that the use of electronic devices in the community made them targets for opportunistic crime. In addition, they were not confident to use the application on the cell phones. The list of questions on the application was also very long and made home visits into more of an administrative exercise, which clients found unhelpful. Questions on TB symptoms came quite late in the process.

The district CHW trainer was busy with other training interventions and was not available to retrain the team in the use of the m-health technology. To avoid a breakdown in records, the team agreed to continue with the paper-based recordkeeping system, but the forms were not made officially available because of the move to the electronic system. The team could also have been trained by the OTL, but because of time constraints and other responsibilities, she was conflicted in her commitments to the team.

In the final month of the project, a review of their daily report register showed that they created a column to record TB contact tracing and identify patients lost to follow-up, but were not recording screening for TB. Community health workers might informally record TB screening in their own diaries, but this was not a reliable or accessible form of data. Community health workers also questioned the collection of such data, as they did not receive feedback on their monthly reports and the data they collected did not seem to inform future actions.

The OTL also had a series of planned campaigns and events with the team, which included TB screening. Although she reported on these events, there was no reliable data on numbers of people screened or referred for TB.

#### Use of referral forms by community health workers

Prior to the QIP, CHWs admitted they referred patients by word of mouth. This was confirmed by the facility manager, who wanted more formal communication with the facility. In their opinion the use of the referral forms improved communication between the CHWs and the facility. They believed this mode of communication strengthened the quality of their referrals.

The provision of feedback using the same referral tools was supported by nurses in the facility because this was necessary to inform the CHWs of follow-up activities and was a data element in the Ideal Clinic norms and standards.

#### Training of community health workers

There were different perceptions on who was responsible for training. One viewpoint was that the district trainer was responsible for coordinating all training of the WBPHCOTs. The OTL then supported experiential ad hoc learning by the CHWs related to specific queries or clients. However, the perception of the subdistrict coordinator was that the OTL was responsible for all training with her team. There was no refresher training on TB, as the district trainer was unable to make time in their programme. The focus was on training workers for coronavirus disease 2019 (COVID-19) vaccine administration. The team mentioned that they had received invites for other scheduled training sessions, but unrelated to TB.

#### Implementation of a multidisciplinary team

The QIP team wanted to develop an MDT to coordinate care between the community-based and facility-based services. The CHWs detailed difficulties that they experienced with responding to queries from clients in the community about health and social services. They felt ill-equipped to address some of the issues and thought that such an MDT meeting would help them. The facility-based services, however, felt understaffed and under pressure of a high workload and unable to commit to MDT meetings.

#### Supportive supervision from outreach team leaders

The OTL believed she could only provide support in short informal sessions with the CHWs to address minor queries. She had to divide her time between two teams across two facilities and was also given duties related to COVID-19 vaccination and school health services. As a result, CHWs had very little to no supervision. The CHWs reported that this negatively impacted the quality of their work. The OTL acknowledged the difficulties with managing two teams and that she was unable to provide supervision during their visits in the community. She also admitted not being able to review the feedback from the nurses.

## Discussion

This QIP did not lead to a measurable improvement in the quality of active TB surveillance. Although most of the structural elements were in place, the team was not complete, and the OTL had multiple conflicting duties. In addition, the introduction of m-health technology was unsuccessful and led to a dysfunctional system for recording the activities of the CHW team. The CHW team was under-supervised by the OTL, and there was confusion as to who was responsible for supporting learning and providing training. The referral and feedback system was supported by the facility, although little used, as this also enabled the TB clinic to request help from the CHWs and aligned with the annual audit of the facility by the Ideal Clinic programme. The facility-based health care workers felt that they were under too much work pressure to participate in MDT meetings to coordinate care and support the CHW team.

The successful implementation and maintenance of active TB surveillance in this context was dependent on the successful implementation of the COPC approach to PHC service delivery. Most of the issues that prevented active TB surveillance were related to the implementation of COPC and not to TB surveillance itself. Many of the issues found in this study have been identified as shortcomings of COPC and WBPHCOTs elsewhere in the country.^32^ After reflecting on these findings, a number of key issues were apparent.

### The need for a paradigm shift

Managers have a role to play in improved implementation of COPC.^36^ Their role is to help delegated authorities in the facilities understand their responsibility, not just as providers of curative services, but as providers who facilitate health and well-being in the population through collaboration with the community and social services. The current service delivery platform in primary health care is very focused on curative services and the ‘practice population’. Managers are disinclined to invest time developing services to cater to the needs of the ‘population at-risk’. This is seen in the attitude of the facility manager and nurses towards MDT support for the CHW team and participation in the QIP team. This differs from study 1, where programme managers implied the resources to implement active surveillance were not available. It can be inferred from these findings that more than the lack of resources was the service managers’ disinclination to take responsibility for the community-oriented approach to providing community-based services.

A paradigm shift is needed to fully embrace and embody the COPC approach in service delivery. The benefit of this new mindset will mainly be downstream, with reduced morbidity in the population, a reduction in the catastrophic effects of TB disease and a reduced incidence of TB in the population. Even though the NDoH has adopted a COPC approach, managers still struggle to align their ideals to this policy. Thus, managers in this context prioritised the use of the OTL in other areas outside her primary role. The COPC is at the heart of the NDoH’s initiative in re-engineering primary health care to achieve universal health coverage.^37^ In contexts where COPC has been implemented to address the health care needs of the population, health indicators are known to improve remarkably.^38^ Ultimately, the effect of elevating COPC as a model of care will improve the chances of implementing active surveillance for TB.

### Supportive supervision in community-based services

The manager added to the list of responsibilities for the OTL, but these were not accomplished because she was spread too thinly between them. She prioritised other roles to her role as an OTL. The result was that the CHWs were demoralised because they were unsupported and disregarded. The importance of supportive supervision of CHW teams has been observed elsewhere.^39^ There is growing evidence across SA to show that failed leadership is a barrier to the implementation of WBPHCOTs.^32,40^ The NDoH needs to consider a framework where supervision of the CHW teams is independent of other roles in the facility.^41^ This will create opportunities for the OTLs to provide adequate supervision, coordination, capacity building, and clinical support to the CHW teams.^42^ Outreach team leaders themselves require ongoing support from management in the district and supervision of their own duties. Their functioning reflects on the quality of leadership provided to the community-based services as an integrated service on the district health platform.^43^

### Resource allocation in the community-based services

Resources allocated to community-based services, should be used for this purpose, and not re-allocated to other services and priorities. The failure to recognise the essential role played by community-based TB screening services in the TB epidemic limits its contribution to achieving the goals of the END TB strategy. The poorly constituted team of three CHWs in this context limited the reach of the team within the community. The system requires adequate constitution of the teams to enable them to function optimally, especially in addressing the social determinants of health.^44^ The policy recommends at least six CHWs in a team.^17^ In addition, there should be environmental officers, health promoters, and other stakeholders that assist with the provision of adequate preventive care.^45^ The relationship of this team with the facility was somewhat strained because of the reluctance of the facility-based staff to support them when they felt over-burdened by a high workload. Community health workers also had no bags to carry equipment and provide simple services such as checking blood pressure or weight. This limited their acceptability within the community as patients wanted to see a tangible service and were not happy with just answering questions. There are studies that affirm that these practical activities improve acceptability of their services.^39^

### Digital technology for community health workers

The attempt of the DHS to provide mHealth technology to this team for data collection failed. It can be inferred that this complex intervention required engagement with managers and CHWs alike. A seemingly laudable idea failed because of a poor understanding of the threats to implementing this idea in this context. Introducing digital technology is a complex process. Literature suggests implementation can be assessed in seven domains: clinical condition/co-morbidities, technology, value proposition, adopters (who is using the technology), organisation, wider system, and embedding or adaptation over time.^46^ Reviewing the implementation of this technology using these seven domains, the OTL and the CHWs in the team (the adopters) were worried about the security challenges, and their description of the software (the technology) suggested it was not fit for purpose and frustrated the CHW-client relationship (value proposition). The CHWs also had poor knowledge of technology and lacked insight into how to use the software when it extended beyond what they considered beneficial to their clients. Also, the introduction of the technology was not promoted extensively within the team (embedding and adaptation). They were provided with the devices with no indication as to how the district coordinators continuously followed up on the usefulness of these devices in the community-based services (organisation and wider system). There are many potential advantages to the use of a functioning mHealth system for designing interventions specific to the communities served.^47^

### Health information technologies in the DHS

The WHO asserts that digital health interventions must add value to the quality of services and not just be used for the sake of introducing something novel.^48,49^ Health information systems (HIS) are a recognised factor that impacts the quality of care in community-based services.^50^ Even though the introduction of digital technology failed, where it has been introduced successfully, the technology can contribute to improved performance and outcomes.^51^ It also improves the capacity of managers to monitor and evaluate processes and outcomes. The paper-based system is often inadequate for recordkeeping purposes, as observed in this project. The use of technology allows collation of data that is easy to analyse.^49,51^ This study showed how these systems could very easily become obsolete when there is inadequate commitment to the processes of implementation. Addressing this challenge will require training for both managers and the WBPHCOTs from the outset on the use of health information and mHealth technologies.^52^ When managers are comfortable with the use of these systems in recordkeeping for information purposes during their analysis of community-based activities, the information is easier to use for designing innovative interventions. Reports are also easier to compile, and managers are able to record how their interventions have been adapted to the needs of their communities.

### Strengths and limitations of the study

The inclusion of the CHWs in the QIP team was a strength of this study. Their inclusion in the decision-making strengthened the QIP process and gave them a voice. The absence of the subdistrict coordinator, facility manager and nurses from the team made it more difficult to implement changes within the facility and denied the team an important perspective. The inclusion of other community stakeholders could have strengthened the QIP. Community leaders were approached but did not commit to join the QIP. Although the coordinator of community-based services was not included in the team, her reflection on the findings helped us understand the managerial decisions that impacted team functioning in this context. The effect of the COVID-19 pandemic was still evident as CHWs who could have been part of the WBPHCOT were employed to do other activities related to screening patients in the facility and organising clients for vaccination.

The transferability of these findings has been ensured by providing an in-depth description of the context, including the relevant stakeholders in this QI cycle. The researcher provides a description of the processes, and the team reflections and key issues in the findings have been highlighted based on the team’s perspective. Furthermore, the reflections of the subdistrict coordinator were provided to give depth to the description of resource constraints and challenges faced with managerial decision-making. The interview of the OTL separate from the team was indicated to see how the OTL perceived the challenges experienced and her role on the team.

## Implications and recommendations

The following recommendations can be made from the findings:

### District managers and coordinators of ward based primary health care outreach teams

- Managers and all members of the PHC team need to share a common understanding of COPC and be committed to implementing the principles. The team is not an extension of the facility-based services but an important tool to provide the community-based services within a COPC approach. Leadership and training are needed to enable this change in mindset.
- The district health services (DHS) needs to commit to properly constituting teams in relation to the numbers needed for the population.
- The use of health information technologies is essential to the development of the services in this context. The implementation of digital technologies to record data in the community and integrate it into the HIS requires a champion who is keen to engage with staff and address challenges.

### Facility managers and outreach team leaders

- The facility manager needs to coordinate human resources in the facility to provide support to the team through an MDT.
- Referrals from the WBPHCOT should be actively managed and feedback provided to the team on a regular basis to encourage growth within the team.
- A community health forum should be developed and can play an important role in community engagement, especially as it is relevant to TB screening activities.
- The OTL must be available, dedicated, and provide supportive supervision to the CHW teams and should not be conflicted by other duties in the facility or services.
- The CHWs should provide community-based services and not serve as back-up for short-staffed primary care facilities.
- The responsibilities for workplace-based learning and more formal training need to be clarified within the district health services; the OTL is responsible for training in the WBPHCOT.

### Future research

- Further QIPs are needed to incrementally monitor and improve the quality of active TB surveillance, and the QIP team should involve all the key role players.
- More research is required for a feasible and reliable system of collecting data and reporting on active surveillance for TB in this context.
- Further research is required to investigate supportive supervision in community-based health services and set measurable outputs.
- Another area for further research is on how to improve coordination of services between facility- and community-based members of the primary health care team.

## Conclusion

No improvement in active surveillance for TB was seen, despite the capability and motivation of the CHWs. There was a facility-centred focus to service delivery, which negated a community-oriented model of care. Community health worker teams were unable to perform active surveillance for TB because of poor record systems, poor implementation of mHealth technology, a lack of supportive supervision, a failure of workplace-based training, an absence of support for their work from the facility-based staff, and inadequate resources. Active surveillance for TB, along with many other important community-based services, depends on a paradigm shift in the managers that leads to a shift in allocation of resources and attention to the factors listed earlier. The introduction of WBPHCOTs has huge potential but appears to be failing because of a lack of commitment to community-oriented primary care.
